# Regulation of MicroRNA-155 in Atherosclerotic Inflammatory Responses by Targeting MAP3K10

**DOI:** 10.1371/journal.pone.0046551

**Published:** 2012-11-26

**Authors:** Jianhua Zhu, Ting Chen, Lin Yang, Zhoubin Li, Mei Mei Wong, Xiaoye Zheng, Xiaoping Pan, Li Zhang, Hui Yan

**Affiliations:** 1 Department of Cardiology, First Affiliated Hospital, School of Medicine, Zhejiang University, Hangzhou, People's Republic of China; 2 Department of Infectious Disease, First Affiliated Hospital, School of Medicine, Zhejiang University, Hangzhou, People's Republic of China; 3 Department of Cardiothoracic Surgery, First Affiliated Hospital, School of Medicine, Zhejiang University, Hangzhou, People's Republic of China; 4 Cardiovascular Division, School of Medicine, King's College London, London, United Kingdom; University of Barcelona, Spain

## Abstract

**Aims:**

Accumulating evidence suggest that numerous microRNAs (miRNAs) play important roles in cell proliferation, apoptosis, and differentiation, as well as various diseases that accompany inflammatory responses. Inflammation is known to be a major contributor to atherogenesis. Previous studies provide promising evidence in support of the role of miRNAs in cardiovascular disease. However, mechanistic data on these small molecules in atherosclerosis (AS) are still missing. The present study aims to investigate the potential role of miRNAs in AS.

**Methods and Results:**

The miRNA transcriptase was verified by TaqMan real-time polymerase chain reaction assay. Thoracic aorta samples were obtained from Apolipoprotein E knockout mice, and plasma samples were from coronary artery disease (CAD) patients. The results showed that the miR-155 level was the most significantly elevated both in AS mice and CAD patients relative to the normal control. The functional role of miR-155 in the atherosclerotic path physiological process was also observed in vivo and in vitro. The observations suggested that miR-155 is a part of a negative feedback loop, which down-modulates inflammatory cytokine production and decreases AS progression. miR-155 was also found to mediate the inflammatory response and mitogen-activated protein kinase (MAPK) pathway by targeting mitogen-activated protein kinase kinase kinase 10.

**Conclusions:**

miR-155 contributes to the prevention of AS development and progression. It may also be involved in the posttranscriptional regulation of the inflammatory response and MAPK pathway by targeting mitogen-activated protein kinase kinase kinase 10.

## Introduction

Atherosclerosis (AS) is a multifactorial disease driven, in part, by chronic inflammation in response to cholesterol accumulation. The pathogenesis of AS in relation to immuno-inflammatory responses, oxidative stress, apoptosis, hemodynamic changes, etc., is an extremely complex pathological process. Inflammation is recognized as a major contributor to atherogenesis [1]. These effects are mediated by the components of the innate immune system (macrophages and dendritic cells, DCs) and of the adaptive immune system (T lymphocytes) [2]. Up to now, little is known regarding the complex upstream regulators of gene expression and translation involved in the responses to AS.

MicroRNAs (miRNAs) are a novel class of short (∼22 nucleotides) non-coding RNAs. They have been identified as important post-transcriptional inhibitors of gene expression via base pairing with the 3′ untranslated regions (UTRs) of mRNAs and promoting mRNA instability [3]. Recent studies have uncovered the profound and unexpected roles of miRNAs in controlling the diverse functions of cardiovascular sciences [4]–[7]. miRNAs are indeed likely to become an integral part of our fundamental comprehension of AS. Moreover, miR-155 is highly expressed in activated B cells, T cells, macrophages, and DCs [8]. It is up-regulated in primary murine macrophage and oxidized low density lipoprotein (oxLDL)-stimulated monocytes [9], [10]. miR-424 modulates monocyte differentiation and function [11]. miR-17-5p-20a-106a controls monocytopoiesis through the regulation of AML1 and M-CSF receptors [12]. miR-146 is induced in macrophages by several microbial components and proinflammatory cytokines in an NF-kB-dependent manner [13]. In addition, the miR-17-92 cluster and miR-150 regulate B cell development [14], [15]. The myeloid-specific miR-223 regulates progenitor proliferation as well as granulocyte differentiation and activation during inflammation [16]. All miRNA-dependent regulators of immune cells are involved in the control of vascular inflammation and AS. They are interesting candidates that may also be involved in immuno-inflammatory responses during AS.

Previous studies provide promising evidence in support of the role of miRNAs in cardiovascular disease. However, mechanistic data on these small molecules in AS are still missing. The associated functional role of miRNAs in animal models with minimal confounding factors in conjunction with human samples is also unclear. Hence, the present study was designed to shed light on these issues. The potential role of miRNA on atherosclerotic immuno-inflammatory responses is also investigated.

## Results

### Detection of miRNAs specifically expressed in atherosclerotic mice

Based on the findings from our previous study that some miRNAs were up-regulated by oxLDL-treated human primary monocytes and on a survey of previously reported miRNA profiling results [10], [17]–[19], five miRNAs (miR-155, miR-146a, miR-125a-5p, miR-29a, and miR-9) were selected in the present study. These miRNAs are specifically expressed in the immuno-inflammatory response of AS. Real-time PCR was performed to analyze miRNA levels in vascular tissues and bone marrow-derived mononuclear cells (BMMCs) from normal and ApoE knockdown mice. The results confirmed the expression levels of all five selected miRNAs in vessels and BMMCs were significantly higher in ApoE knockout mice than that in normal mice (Fig. S1).

### Evaluation of miRNAs in plasma and PBMC as new biomarkers for CAD

**Figure 1:**
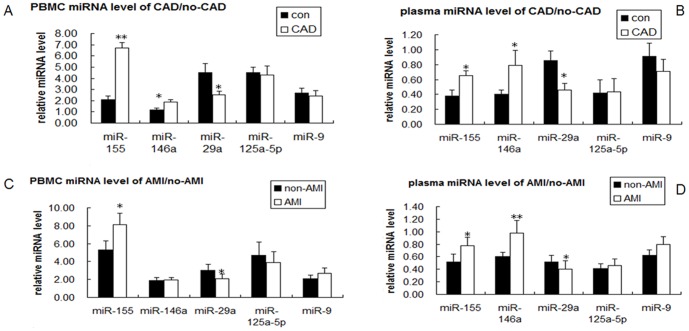
Relative expression levels of specified miRNAs in PBMCs and plasma from CAD patients. PBMC (A) and plasma (B) were collected from patients with angina-like symptoms but safely excluded by coronary angiogram (*n* = 22), and from CAD patients who had at least one diseased vessel (*n* = 65). (C) and (D) PBMC and plasma were collected from patients suffering from AMI (*n* = 30), and patients with coronary heart disease but without AMI (*n* = 35). Total RNA was isolated, reverse-transcribed, and subjected to real-time PCR analysis. Data are represented as mean ± SD; *P<0.05 and ** *P*<0.01.

To further investigate the characteristics of miRNAs as potential biomarkers of CAD, 87 male subjects were studied. TaqMan real-time PCR analysis was performed to detect the m RNA level of miRNAs, All the 87 patients with chest pain and after angiographic documentation were selected as the study cohort. Of these 87 patients, 30 were AMI patients, 35 were non-AMI, and 22 were without evidence for CAD. The clinical characteristics of the 2 study populations are summarized in Table SI. As shown in Fig. 1, compared with the control group, miR-155 and miR-146a were significantly induced in the CAD group both in PBMC (Fig. 1A; 219.05%, *P*<0.01; 59.75%, *P*<0.05) and plasma (Fig. 1B; 71.05%, *P*<0.05; 97.5%, *P*<0.05), respectively. In contrast, miR-29a was reduced both in PBMC (Fig. 1A; 43.58%, *P*<0.05) and plasma (Fig. 1B; 45.8%, *P*<0.05). Furthermore, the expression of the selected miRNAs between the non-AMI and AMI patients were compared. miR-155 was found to be slightly induced in the AMI group both in PBMC (52.8%, *P*<0.05) and plasma (50%, *P*<0.05). miR-146a was increased only in plasma (63.33%, *P*<0.01). miR-29a was still decreased in both plasma (23%, *P*<0.05) and PBMC (30%, *P*<0.05). No difference was observed in miR-9 and miR-125a-5p. (Figs. 1C and 1D).

### Regulation of inflammatory cytokine secretion by miR-155 in vitro and in vivo

**Figure 2:**
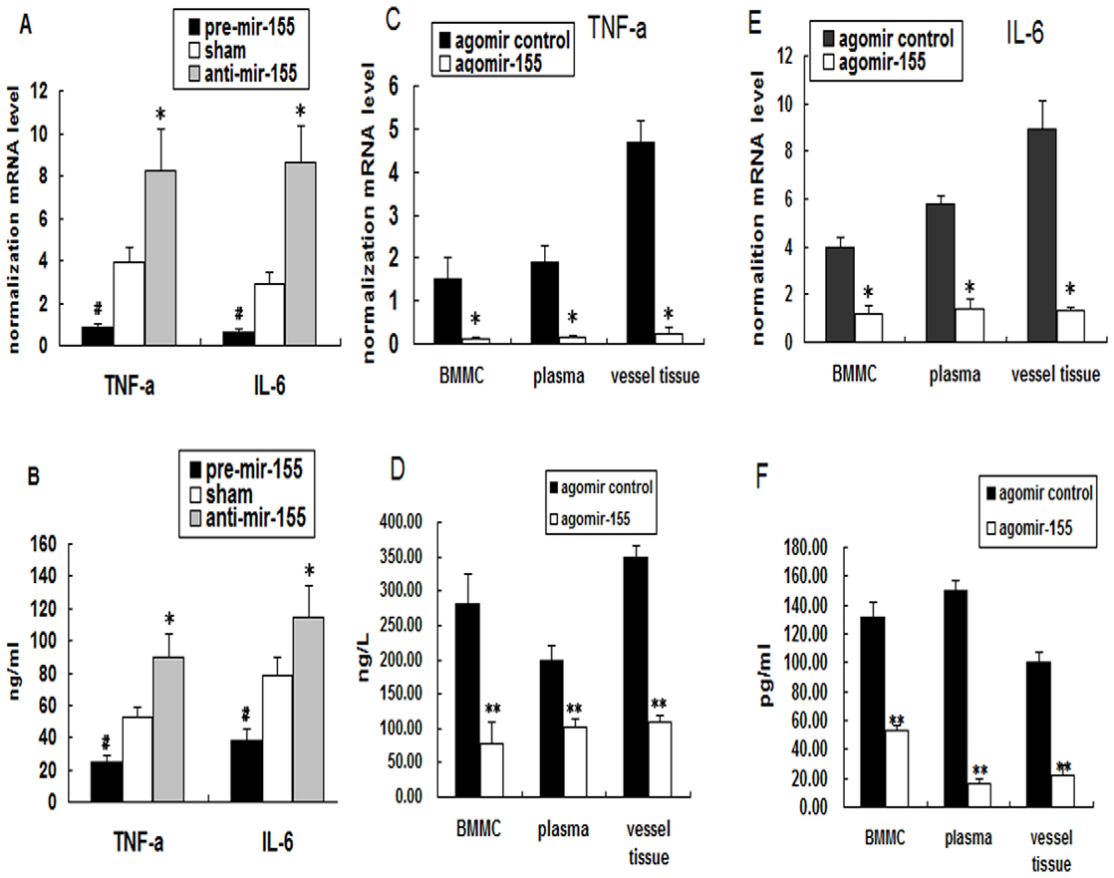
Effects of miR-155 on inflammatory cytokine secretion. PMA-induced THP-1 cells were transfected with miRNA mimic/inhibitor, and then incubated with oxLDL for 24 h. TNF-α and IL-6 expression levels were regulated after miR-155 mimic and inhibitor incubation. (A) The relative mRNA levels of TNF-α and IL-6. miR-155 was over-expressed via a single tail-vein injection of cholesterol-modified agomiR-155 into ApoE^−/−^ mice. (B) ELISA detection of the protein levels of TNF-α and IL-6. (C) and (E) TaqMan RT-PCR was used to analyze the levels of IL-6 and TNF-α expression in vascular tissues, plasma, and BMMCsAnalysis using ELISA detected the protein levels of IL-6 and TNF-α in vascular tissues, plasma, and BMMCs in ApoE^−/−^ mice (D) and (F) compared with the agomiR control. Mean ± SD calculated from five different experiments; *P<0.05 versus the sham or agomiR control group, **P<0.01 versus the “agomiR control” group.

To investigate the possible role of miR-155 in the inflammatory response of AS, the chemically synthesized miR-155 inhibitor or mimic was used, the tranfect efficiency was shown as FigureS2. As shown in Figs. 2A and 2B, the miR-155 mimic decreased some of the inflammatory cytokine (IL-6 and TNF-α) secretions of oxLDL-induced macrophages. On the other hand, the miR-155 inhibitor increased their secretions both in the protein and mRNA levels. This result indicated that miR-155 transfection affects the inflammatory response of oxLDL-treated macrophages.

To elucidate further the in vivo effects of miR-155 on inflammatory response, miR-155 was over-expressed via a single tail-vein injection of cholesterol-modified agomiR-155. Three days after the administration of agomiR-155, TaqMan reverse transcriptase (RT)-PCR analysis showed a dramatic induction of miR-155 expression in vascular tissues, plasma, and BMMCs (Fig. S3). ELISA analysis confirmed the associated decrease in the protein levels of IL-6 and TNF-α in vascular tissues, plasma, and BMMCs (Figs. 2C and 2E) compared with the agomiR control. TaqMan RT-PCR analysis also showed a dramatic reduction in IL-6 and TNF-α expression (Figs. 2D and 2F).

### Prevention of in vivo AS development and progression by miR-155 overexpression

**Figure 3:**
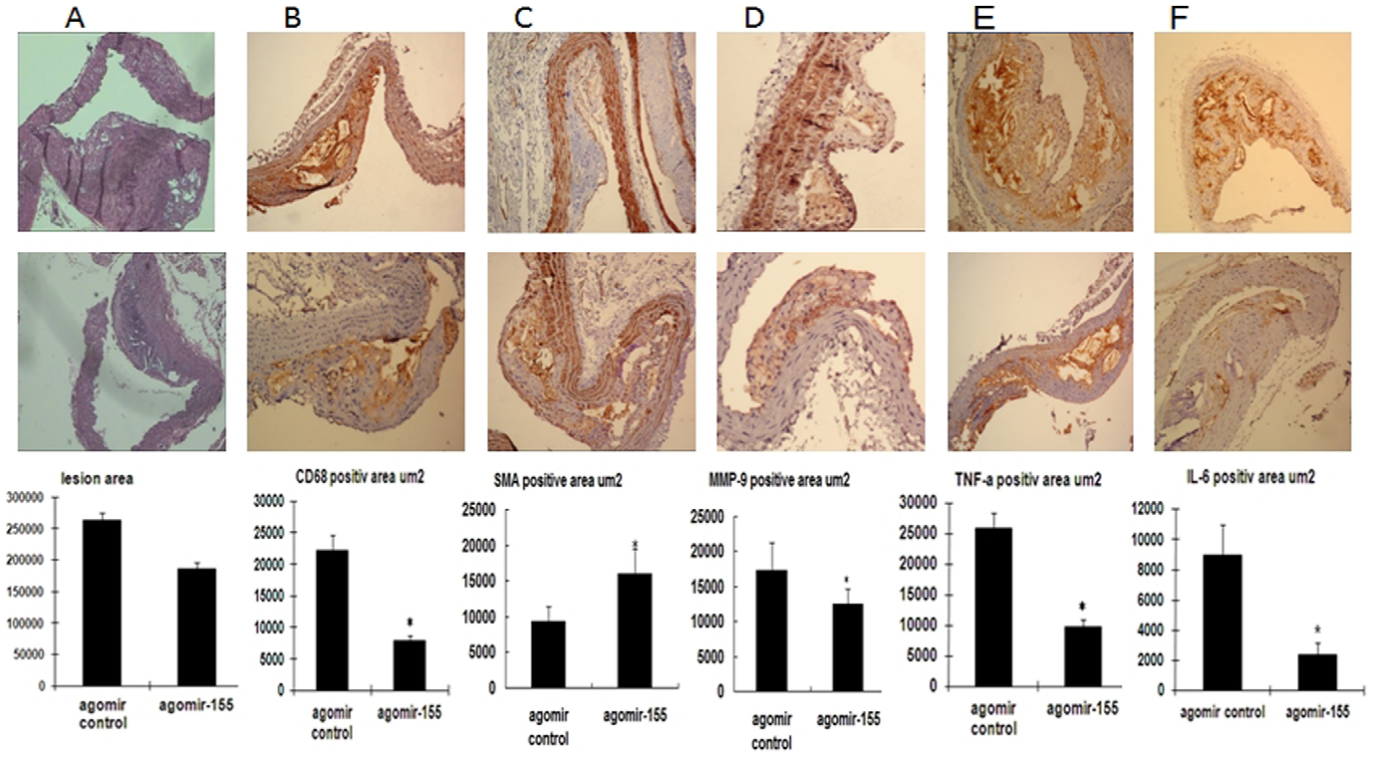
miR-155 suppresses atherosclerotic plaque progression, reduces macrophage accumulation, as well as decreases TNF-a and IL-6 levels in the thoracic aorta of ApoE^−/−^ mice. (A) Paraffin-embedded cross sections from agomiR control or agomiR-155-infused ApoE^−/−^ mice were obtained throughout the thoracic aorta area and stained with hemolysin and eosin (HE). The sections were then imaged and quantified with Image-Pro Plus at a 400× magnification. (B) agomiR-155 reduces macrophage accumulation and MMP-9 expression, induces smoothe muscle cell composition (B,C, D) as well as decreases the TNF-a and IL-6 levels (C) within atherosclerotic plaques. Paraffin-embedded serial sections were stained with HE, rat anti-mouse CD68 antibody, and rat non-specific IgG (B) or rabbit anti-mouse TNF-a and IL-6 antibody, as well as rabbit non-specific IgG, respectively (C) and (D), at a 400× magnification. The aortic lesion size as well as CD68-, TNF-a-, and IL-6-positive lesion areas are shown from the agomiR control-infused group or agomiR-155-infused group. Data are represented as mean ± SD (*n* = 12). *P<0.05 compared with the agomiR control group.

To evaluate further the biological role of miR-155 up-regulation on AS development and progression, miR-155 was over-expressed via tail-vein injection of cholesterol-modified agomiR-155. Plasma cholesterol levels were detected. A modest reduction in the levels of TC and LDL (TableS2) in mice treated with agomiR-155 was found compared with the agomiR control group. However, the plasma TG and HDL levels did not differ between groups. Quantitative computer-assisted image analysis showed a slight decrease (about 29.24%) in the extent of atherosclerotic lesions in agomiR-155-treated thoracic aorta, but there was no significant difference (*P*>0.05) (Fig. 3A). On the other hand, the plaque area staining positive for macrophages (CD68) in agomiR-155-infused mice decreased by 64.2% compared with the control (7929±1896 µm^2^ versus 22146±4563 µm^2^; Fig. 3B), the MMP-9 level was decreased by 27.4% (17228±3516 µm^2^ versus 12507±2525 µm^2^) and the SMA level was increased by 29.3% (11330±1987 µm^2^ versus 16032±3122 µm^2^). In addition, TNF-a and IL-6 immunopositive plaque areas also decreased by 62.2% (25896±2512 µm^2^ versus 9781±1122 µm^2^) and 73% (8930±2026 µm^2^ versus 2411±799 µm^2^) respectively, in agomiR-155-infused mice compared with the control (*P*<0.05; Figs. 3C and 3D). These data demonstrated that miR-155 maybe reduce atherosclerotic plaque progression consistent with an anti-inflammatory effect. Hence, the critical protective role of miR-155 in atherogenesis is revealed.

### miR-155 inhibits the MAPK pathway in oxLDL-induced macrophages and ApoE knockdown mice

**Figure 4:**
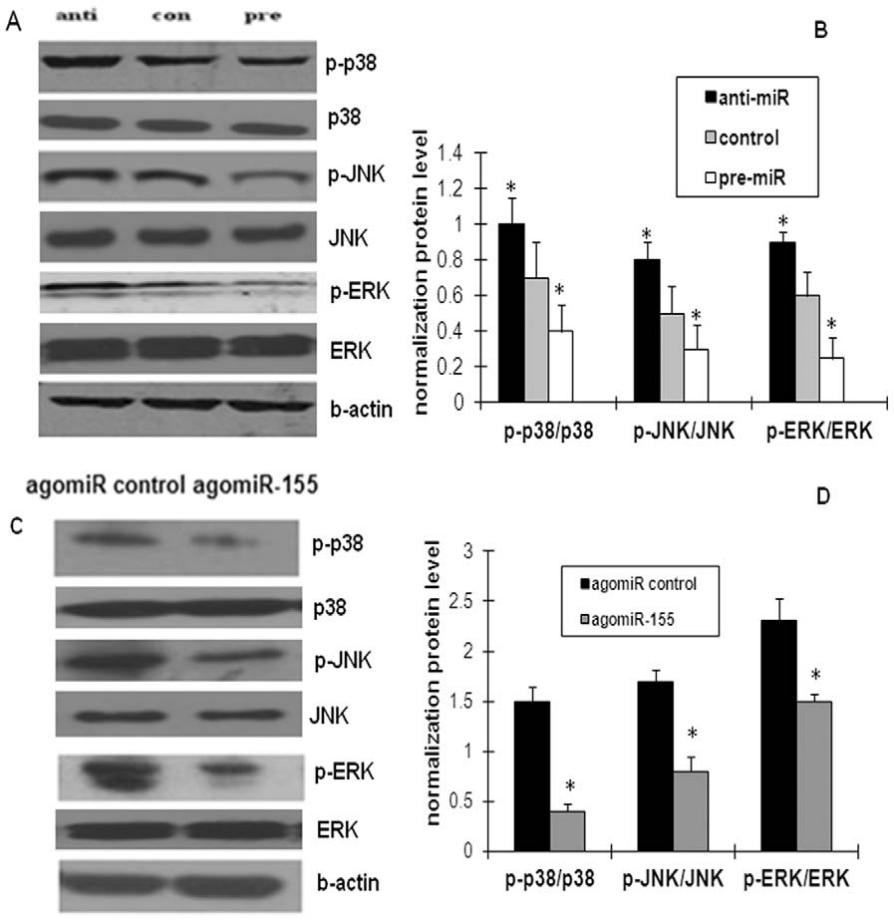
Effects of miR-155 on the activity of the MAPK signaling pathways in oxLDL-stimulated macrophages and in the thoracic aorta of ApoE^−/−^ mice. PMA-induced THP-1 cells were incubated with oxLDL for 24 h, then were transfected with miRNA mimic/inhibitor for another 24 h. The AgomiR control and agomiR-155 were injected into ApoE^−/−^ mice. The phosphorylation of the ERK1/2, p38, and JNK pathways were assayed by Western blot (A). Densitometry ratios of phosphorylation and total level of ERK1/2, p38, and JNK. (B) Band densities measured by Quantity One 1D software. (C) and (D) The protein levels of the ERK1/2, p38, and JNK pathways in the thoracic aorta of ApoE^−/−^ mice. Data (mean ± SD) were from three independent experiments; *P<0.05 compared with the control group.

Maurizio et al. [20] reported that the p38 MAPK pathway is involved in miR-155 inhibition. Some studies also suggest that the JNK pathway is involved in the up-regulation of miR-155 expression in response to poly(I:C) or TNF-α. Therefore, the involvement of the MAPK pathway in the anti-inflammatory effect of miR-155 was interesting to determine. The results showed that the miR-155 inhibitor significantly up-regulated, and its mimic down-regulated the p38, ERK1/2, and JNK phosphorylation pathways stimulated by oxLDL in macrophages (Figs. 4A and B). Moreover, the overexpression of miR-155 inhibited the activation of p38, ERK1/2, and JNK in vivo (Figs. 4C and 4D). Hence, the p38, ERK1/2, and JNK signaling pathways are required to enable the effects of miR-155.

### MAP3K10 is a direct target of miR-155

**Figure 5:**
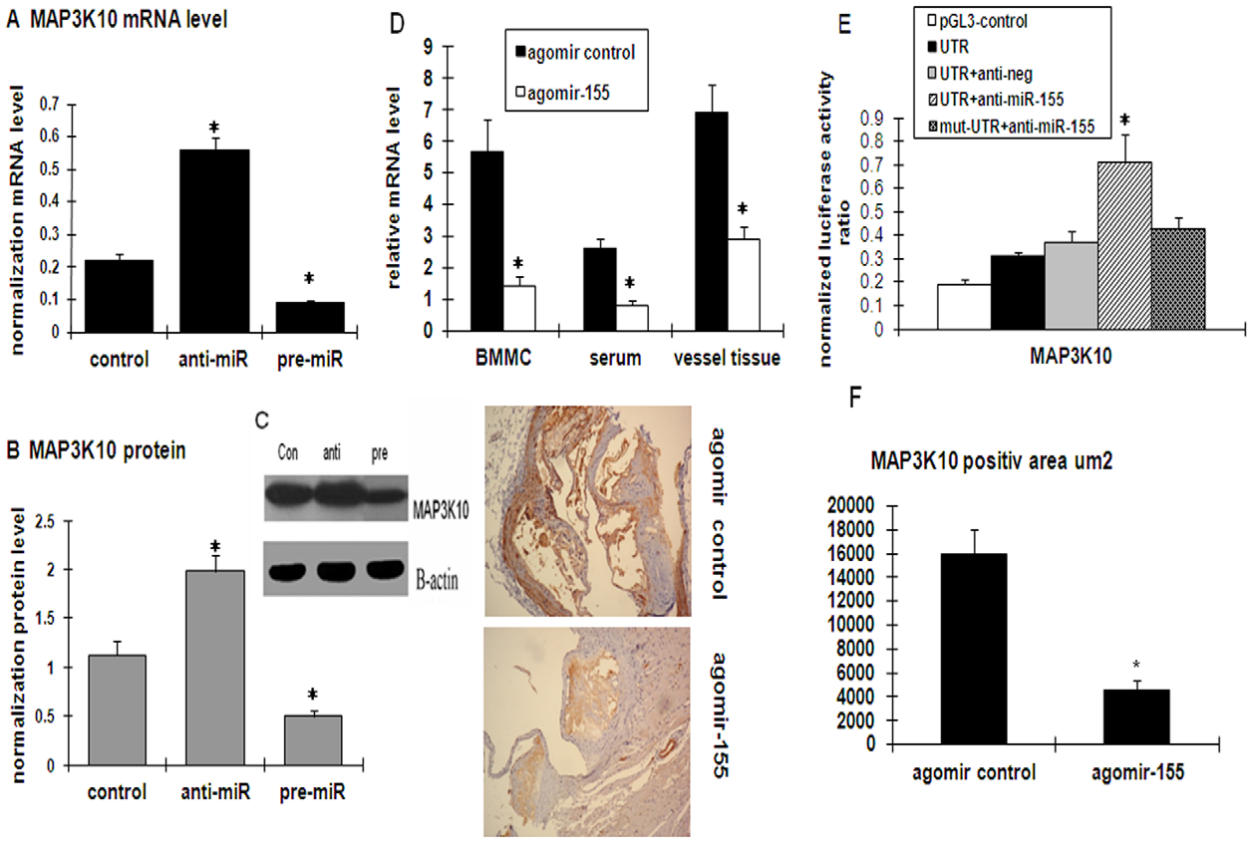
Verification of the potential target genes (MAP3K10) of miR-155 by luciferase reporter assay coincidental with mRNA and protein expression levels. (A) Effects of miR-155 on the transcript level of MAP3K10 in cultured THP-1 cells. (B) The Western blot results of MAP3K10 expression and respective densitometric measurement results in the THP-1 cells are shown. The band density of native cells was defined as the control. Data were obtained from four independent experiments. *P<0.05 versus the control group. (C) the relative MAP3K10 m RNA level was measured by real-time in vessel tissues, plasma and BMMCs of ApoE^−/−^ mice after treatment with agomir control and agomir-155. (D) Immunohistochemical analyses of MAP3K10 expression in ApoE-deficient mice aortic lesion after treatment with the agomiR control and agomiR-155. Data (mean ± SD) were from four independent experiments. *P<0.05 compared with the agomiR control group. (E) THP-1 cells were cotransfected with p- MAP3K10-UTR, p-mut-MAP3K10-UTR, pGL-3 control, anti-neg, and anti-miR-155. Luciferase values were normalized by the Renilla control's luciferase activity. The ratio of luciferase activity of each construct was calculated by a luminometer. Data (mean ± SD) are from four independent experiments. *P<0.01 compared with the pGL3-MAP3K10-3′UTR plasmids only group.

To elucidate the potential mechanism of miR-155 in the regulation of the AS inflammatory response, the putative targets of miR-155 were first identified. Bioinformatics tools in multiple databases (targetscan4.1, PicTar, and miRanda) were used to identify candidates. These tools gave evidence that MAP3K10 is the possible target gene of miR-155. To determine if miR-155 specifically attenuates MAP3K10 expression, the endogenous MAP3K10 mRNA levels were measured through qPCR after the transfection of miR-155 inhibitor or mimic in PMA-induced THP-1. The inhibition of miR-155 expression with miR-155 inhibitor increased, whereas miR-155 expression with miR-155 mimic decreased MAP3K10 mRNA levels relative to those of the control cells transfected with a non-specific miRNA (Fig. 5B). Consistent with these changes in mRNA levels, the level of MAP3K10 protein was also altered by the miR-155 inhibitor and mimic (Figs. 5C and 5D). To observe the in vivo relationship of miR-155 and MAP3K10, changes in the MAP3K10 levels in agomiR-155-injected ApoE knockdown mice were analyzed and compared with the the agomiR control. As expected, the MAP3K10 mRNA levels in plasma, vessel tissues, and BMMC substantially decreased in agomiR-155-injected mice compared with the control group (Fig. 5G). Immunohistochemistry revealed that the administration of agomiR-155, but not agomiR control, was associated with greatly decreased levels of MAP3K10 in vessels (Figs. 5E, 5F, and 5H).

To determine if the regulation of MAP3K10 by miR-155 was specifically mediated by the miRNA mechanism, the 3′-UTR of the MAP3K10 gene containing the miR-155 recognition site was cloned by inserting it downstream to a luciferase reporter. The reporter gene assay showed that compared with the pGL3-MAP3K10-3UTR plasmid cotransfected cells, there was a statistically significant increase and decrease in the activity of the cells cotransfected with the miR-155 inhibitor and mimic, respectively (Fig. 5A, *P*<0.01). This result suggested that miR-155 directly targets MAP3K10.

### MAP3K10 as a functional gene target involved in the miR-155-mediated inflammatory effect

**Figure 6:**
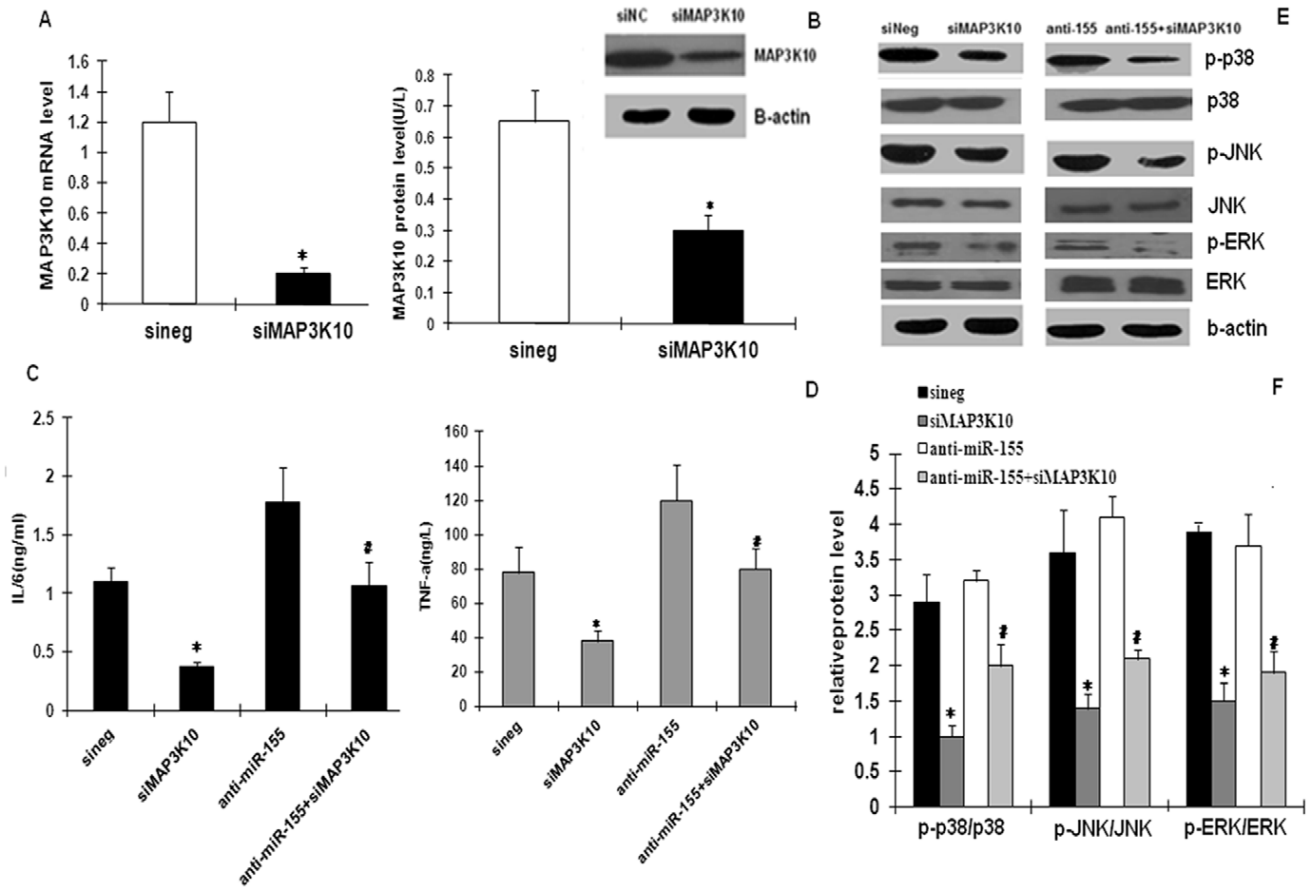
MAP3K10 is a functional target gene involved in the miR-155-mediated inflammatory effect on oxLDL-induced macrophages. (A) MAP3K10 mRNA expression after transfection of small interference RNAs for MAP3K10 (siMAP3K10) or an insignificant control sequence (siNS) in oxLDL-induced macrophages as detected by RT-qPCR. (B) MAP3K10 protein expression detected by Western blot and quantification of Western blot. TNF-α and IL-6 protein levels were assessed by ELISA (C) and (D), as well as the phosphorylation and the total of the ERK1/2, p38, and JNK pathways measured by Western blot in siMAP3K10- or siNS-transfected cells (E) and (F). The miR-155 inhibitor-mediated inflammatory effect was rescued in oxLDL-induced macrophages via small interference RNAs for MAP3K10 (C), (D), (E), and (F). Data are presented as mean ± SD, *n* = 3; *P<0.05 compared with the siRNA control and ^#^ *P*<0.05 compared with the pure miR-155 inhibitor group.

Given the evidence of MAP3K10 regulation by miR-155 at the levels of both RNA and protein presented above, and considering the reported inflammatory effect of MAP3K10, we speculated that MAP3K10 could be a functionally important target of miR-155. To investigate the biological importance of MAP3K10 as a target of miR-155, PMA-induced THP-1 were depleted of MAP3K10 protein and incubation with oxLDL. The effect of miR-155 inhibition was then assayed. The knockdown of MAP3K10 expression via siRNA treatment efficiently repressed MAP3K10 mRNA and protein levels (Figs. 6A and 6B). On the other hand, inflammatory cytokine (TNF-a and IL-6) secretion was decreased (Figs. 6C and 6D), and the p38, ERK1/2, and JNK phosphorylation pathways were down-regulated (Figs. 6E and 6F). These findings are similar with the effects of the miR-155 mimic on oxLDL-treated macrophages. Furthermore, the miR-155 inhibitor-mediated effect on the inflammatory response was counteracted through the inhibition of MAP3K10 by siMAP3K10 on oxLDL-stimulated macrophages (Figs. 6C–6F). Therefore, the data suggested the essential role for MAP3K10 as a mediator of the biological effects of miR-155 on oxLDL-treated macrophages.

## Discussion

Microarrays has been previously performed by our group to analyze the miRNA expression profiles in oxLDL-stimulated human primary peripheral blood monocytes and DCs. Some miRNAs (miR-125a-5p, miR-9, miR-146a, miR-29a, and miR-155) were aberrantly expressed after oxLDL treatment. Consistent with the microarray result, Huang et al. [21] revealed that miR-155 expression was significantly up-regulated in oxLDL-activated THP-1 cells. In the present study, the RT-PCR assay confirmed that miR-155, miR-146a, and miR-9 were aberrantly up-regulated in ApoE knockdown mice. miR-155, miR-146a, and miR-29a were deregulated in patients with CAD. These results provide clues for the future study of their roles in AS.

Among the above miRNAs, miR-155 was significantly up-regulated both in the vessel tissues and mononuclear cells of AS model mice compared with the normal model. This result indicated that miR-155 was induced in the AS model. Recently, identifying the molecular markers correlated with CAD patient typing has attracted much attention. Some studies demonstrated that circulating miRNAs can be detected in the blood and are differentially regulated in patients with CAD, AMI, and heart failure [22]–[25]. In patients with CAD, several miRNAs were also shown to be deregulated in isolated PBMCs [26]. Therefore, the levels of miRNAs were detected both in the plasma and PBMC of patients. As expected, miR-155 expression was found to be increased by about 50%–200% in the CAD patients compared with the healthy control. miR-155 was further induced in the AMI patients compared with the non-AMI patients. miR-155 was found to be strongly induced by a broad range of inflammatory stimuli [8]. Inflammation is also thought to be a major contributor to atherogenesis; hence we believed that the up-regulation of miR-155 in CAD patients contributed to the stressed inflammatory environment of AS and AMI. However, our results were inconsistent with a previous research that revealed miR-155 to be significantly reduced in patients with CAD compared with healthy controls [22]. The reason for this discrepancy is unclear, but the possibile contribution of many confounding factors (e.g., degree of heart damage, ethnic groups, age, and so on) warrant consideration. Worthwhile to mention, to avoid the gender and estrogen effect, all the patients were male, and the control group included relatively healthy individuals who had chest pain but no evidence of CAD by coronary angiogram. The CAD group also included AMI patients. Our results suggested that miR-155 can be a potential marker for predicting the prognosis of CAD patients, moreover, the observed changes in miRNA of PBMCs were more notable than in the plasma, because there are a lot of different kinds of cells in the plasma, but for PBMC, the component partare relative pure, only the mononuclear cells, also this result indicate that miRNAs in PBMC may be sensitive signature for monitoring of cardiovascular diseases.

Recent data have indicated that miR-155, a typical multifunctional miRNA, plays a crucial role in immunity, inflammation and cardiovascular diseases [9]. However, the role of miR-155 in AS inflammatory response has not been systematically studied in depth. The present study revealed for the first time the anti-atherogenic effect of miR-155 both in vitro and in vivo. For the in vivo study, the main obstacle for gene therapy is the process of delivering the genes into the target tissue effectively and safely. Compared with other tissues, vessel tissue is more easily targeted by delivery systems of effective molecules and genes. In vivo gene silencing with miRNA has been reported using both viral vector delivery and high-pressure, high-volume intravenous injection of synthetic miRNAs, but these approaches have limited if any clinical use [27]. Cholesterol-conjugated agomiR was used because of it involves in the cause or pathway of human disease with a clinically acceptable, easier dose control, drug-like properties, and documented high effectiveness in over-expressing target miRNA with long-lasting efficacy under in vivo conditions [27], [28]. In the present study, increased circulating levels miR-155 mildly reduces atherosclerotic burden in ApoE^−/−^ mice. This reduction is associated with a constellation of potential beneficial effects, including decreased expression of proinflammatory cytokines and decreased macrophage accumulation within atherosclerotic lesions. Notably, the reduction in atherosclerotic burden was not statistically significant possibly because of the limited sample size and significant interanimal variability in the measurements.

To study further the potential molecular mechanism of miR-155 therapy in AS, an in silico search of potential targets was performed with the help of currently available bioinformatics. We propose that MAP3K10 may be the target of miR-155 and it was verified by luciferase reporter assay. In addition, MAP3K10 mRNA and protein expression in macrophages and ApoE^−/−^ mice were capable of being regulated by miR-155, as determined by both gain- and loss-of-function approaches.

MAP3K10 is a member of the mixed lineage family of kinases, like other typical inflammatory pathway-related kinases. This kinase functions preferentially in the JNK signaling pathway, which phosphorylates a diverse range of proteins and plays significant roles in cellular proliferation and differentiation, inflammatory and immune responses [29]. The overexpression of MAP3K10 can inhibit endocytic functions [30]. All these functions indicate potential relationships among MAP3K10, atherosclerotic processes, and inflammatory responses. The effects of miR-155 on the inflammatory response and the potential mechanisms between miR-155 and its target MAP3K10 were further explored. miR-155 was found to regulate the release of IL-6 and TNF-α both in vivo and in vitro. MiR-155 regulates cytokines by targeting C/EBPB, which is a positive regulator of IL-6 capable of transcribing a large number of cytokine-encoding genes [31]. Similar results (IL-6, TNF-α) were obtained from LPS-activated DCs after miR-155 knockdown [32] also several other studies showed that miR-155 has a pro-inflammatory role in microglia and is necessary for the progression of the immune response through the modulation of SOCS-1 (suppressor of cytokine signalling 1) an inducible negative feedback inhibitor of JAK/STAT signaling pathway [33], [34] also Ana L. Cardoso found that *F.n.* induces miR-155 expression and leads to down-regulation of SHIP (one of the key regulators the PI3K/Akt pathway), resulting in enhanced pro-inflammatory responses. [35], here we confirmed again with those results, verified that miR-155 can exert a major inhibitory role in fine-tuning the inflammatory response, but the most intriguing founding is the mechanism discover for the relationship between new target MAP3K10 and MAPK pathway and miR-155, meanwhile we use the special model for in the AS pathological process both in vitro and in vivo, further confirmed the positive role of miR-155 in AS inflammatory response.

The MAPK superfamily of serine/threonine kinases has indeed emerged as an important component of cellular signal transduction and MAPKs play a key role in regulating the expression of many proinflammatory genes implicated in the development of AS [36]. Two MAPK cascades (p38 and JNK) had been to reported to be involved in miR-155 regulation [9]. There may be a cross-talk between MAPK pathways and the inflammatory response. Given that miR-155 regulates the expression of inflammatory factors in oxLDL-activated macrophages, we hypothesized that there is an association between the MAPK signaling pathway and the miR-155 effect. In line with this, evidence that the three major classes of MAPKs (ERK1/2, p38, and JNK pathways) were all activated in oxLDL-induced macrophages by the miR-155 inhibitor was provided in the present study. In contrast, these effects were inactivated after the up-regulation of miR-155 expression.

Meanwhile we have found that the miR-155 inhibitor-mediated pro-inflammatory effect was rescued after inhibiting MAP3K10 with siRNA. Interestingly, the down-regulation of MAP3K10 resulted in similar effects as the miR-155 up-regulation in oxLDL-stimulated macrophages. We then concluded that miR-155 can at least partly down-regulate inflammatory responses in oxLDL-induced macrophages, and that MAP3K10 is indeed a functional target gene of miR-155 that is involved in the miR-155-mediated anti-inflammatory effect on oxLDL-stimulated macrophages. Therefore, miR-155 could exert regulatory activity, which would limit the over-production of inflammatory cytokines and the signaling pathways of oxLDL-mediated macrophages. These activities reflect the protective roles that miR-155 may play in AS.

In conclusion, the results of the present study strongly indicate that one major mechanism underlying the anti-atherogenic effect of miR-155 could be an anti-inflammatory effect by targeting MAP3K10. The present results can be the foundations of new therapeutic strategies for the treatment of AS or for the prevention of atherosclerotic immuno-inflammation. In addition, several other miRNAs (such as miR-146a, miR-29a, and miR-9) are also suggested to be deregulated in AS tissues or CAD patients. The correlation of the deregulation of this panel of miRNAs with AS will be the subject of further studies of our group.

## Methods

An expanded Materials and methods section is available in the online supplementary data.

### Cell culture

The human monocytic cell line THP-1 was obtained from the American Type Culture Collection (Rockville, MD, USA). To induce monocyte differentiation to macrophage, THP-1 cells were cultured with 100 nM propylene glycol monomethyl ether acetate (PMA) (Calbiochem, San Diego, CA) for 24 h.

### In vivo assay

C57BL/6J mice were purchased from the Shanghai Institutes for Biological Sciences (Shanghai, China). Apolipoprotein E (ApoE^−/−^) mice was from the BeiJing Xie He Medical College (BeiJing, China). The miR-155 agomiR was chemically modified and cholesterol conjugated. A mismatched miR-155 agomiR was also synthesized as a control. For delivery of cholesterol-conjugated miR-155 agomiR, 10 nmol miR-155 agomiR and mismatched miR-155 agomiR in 0.1 ml saline buffer were tail vein-injected once every 3 d and lasted for 2 weeks, respectively. All animal experiments were conforming to the Guide for the Care and Use of Laboratory Animals published by the US National Institutes of Health (NIH Publication No. 85-23, Revised 1996) and approved by the Animal Care and Ethics Committee of the University of Zhe Jiang.

### Small RNA transfection

The miR-155 mimic and inhibitor were transfected into PMA-induced THP-1 macrophages using the fast transfection protocol recommended for the Hiperfect transfection reagent (Qiagen) at a final concentration of 50 nM. SiMAP3K10 and siNS (not significant) duplexes were synthesized by Dharmacon RNAi Technologies.

### miRNA and mRNA real-time quantitative polymerase chain reaction (PCR)

To detect miRNAs from cells, tissues, and plasma, total RNA was isolated using the miRVana miRNA isolation kit (Ambion) according to the protocol of the manufacturer. The mRNA levels were analyzed using SYBR-GREEN reagent kits on an Applied Biosystems 7000 real-time PCR system.

### Enzyme-linked immunosorbent assay (ELISA) of inflammatory markers

Cell or plasma supernatants were analyzed to determine the presence of TNF-α and IL-6 using Sandwich Enzyme Immunoassay kits (R&D) according to the manufacturer's instructions.

### Western blot analysis

The protein extracts were denatured. The solubilized proteins (20 µg) were subjected to electrophoresis on 10% polyacrylamide sodium dodecyl sulfate gels. The membranes were incubated with primary and secondary antibodies (Abcam). The immunofluorescence signal was detected by an Odyssey infrared imaging system (Li-cor, USA)

### Cloning of the 3′ UTR of MAP3K10 mRNA and the reporter gene assay

The THP-1 cells were cotransfected with a p-MAP3K10 UTR and mut-MAP3K10 UTR miRNA luciferase reporter vector and miR-155 inhibitor using Lipofectamine 2000 (Invitrogen). After 24 h, firefly luciferase activity was determined using the dual-luciferase reporter assay system.

### Analysis of atherosclerotic plaque size and composition

The mice were sacrificed after two weeks injection and feeding another 28 days. Plasma cholesterol was measured with a commercially available cholesterol kit (Sigma). Morph metric and immunohistochemical analyses were performed. The primary antibodies used were anti-mouse CD68 (Santa Cruz), anti-SMA (sigma), anti-MMP-9 (Santa Cruz), anti–MAP3K10 (Sigma), anti–TNF-a (Santa Cruz), and anti–IL-6 antibody (Santa Cruz).

### Study population

Patients with angina pectoris undergoing clinically indicated coronary angiography were consecutively recruited into the current study. Patients with histories of leukopenia, severe hepatic or renal dysfunction, and heart failure, as well as those with evidence of an inflammatory or malignant disease were excluded. The patients were divided into two study groups by coronary angiogram The first is the control group who are relative heathly, consisting of patients who have chest pain, underwent coronary angiography for angina-like symptoms, but for whom coronary artery disease (CAD) was safely excluded by coronary angiogram. Segments were classified as having no significant stenosis (normal, with wall irregularities, or with <50% lumen reduction). The second is the CAD group consisting of patients who had at least one diseased vessel (≥50% stenosis of luminal diameter). Group 2 was further divided into two subgroups. Subgroup 1 was an acute myocardial infarction (AMI) group. The criteria for AMI were typical angina associated with ST segment elevations in an electrocardiogram, and at least three occurrences of elevated plasma creatine kinase and troponin-I. Subgroup 2 was a non-AMI group consisting of patients with angiographically documented CAD but no evidence of AMI. A total of 22 healthy controls and 65 CAD patients (30 AMI and 35 non-AMI) were included in the study population. The study was the conformed with the principles outlined in the Declaration of Helsinki for use of human tissue or subjects and approved by the institutional ethics committee of the Zhejiang University. We also obtained informed written consent from all participants involved in our study.

### Collection and storage of plasma and peripheral blood mononuclear cells

Blood samples for miRNA detection were collected from the patients. The samples were processed within 1 h of collection by two-step centrifugation. Peripheral blood mononuclear cells were isolated from the fresh blood of patients and mice using Ficoll–Hypaque. The supernatant and cells were transferred to RNase/DNase-free tubes and stored at −80°C.

### Statistical analysis

Data are presented as mean ± SD. Statistical comparisons between experimental groups were performed by Student's t tests. All tests were performed two sided, and *P*<0.05 was considered to indicate statistical significance. For all statistical analyses, the statistical software SPSS 17.0 for Windows was used.

## Supporting Information

Figure S1 **The expression of miRNAs in atherosclerosis mice.** Detection of miRNA in vessel and bone-marrow derived mononuclear cell of APOE knockdown mice versus normal mice (n = 5), miRNAs was detected by TaqMan PCR.*P<0.05, **P<0.01.
(TIF)

Figure S2 **The expression of miR-155 after transfection.** Detection the miR-155 expression after transfection with miR-155 mimics and miR-155 inhibitor by TaqMan PCR. (n = 4, *P<0.01, ^#^P<0.01).
(TIF)

Figure S3 **The expression of miR-155 after injection with agomir in vivo.** Detection of the miR-155 level in the BMMC, plasma and thoracic aorta of APOE knockdown mice injected by agomir control and agomir-155 (n = 5), miRNAs was detected by TaqMan PCR. *P<0.01.
(TIF)

Table S1 **Characteristics of study cohort.** TC, total cholesterol; TG, total glyceride; HDL, high-density lipoprotein; LDL, low-density lipoprotein; WBC, white blood cell; P1: comparison between patients with AMI and without AMI. P2: comparison among patients without and with CHD, Data are presented as means (±SD) or number (%).
(DOCX)

Table S2 Plasma Cholesterol Levels of APOE-/- mice injected with agomir control and agomir-155 *P<0.05 n = 12.
(DOCX)

File S1 **Supplementary methods and figures.**
(DOC)
