# Correction: Regulation of MicroRNA-155 in Atherosclerotic Inflammatory Responses by Targeting MAP3K10

**DOI:** 10.1371/annotation/c57231bc-e3fb-4703-8ba7-d3d2841c479a

**Published:** 2013-03-20

**Authors:** Jianhua Zhu, Ting Chen, Lin Yang, Zhoubin Li, Mei Mei Wong, Xiaoye Zheng, Xiaoping Pan, Li Zhang, Hui Yan

Authors Jianhua Zhu and Ting Chen should have been noted as having contributed equally.

Authors Li Zhang and Hui Yan should have been noted as corresponding authors, with the email addresses li.zhang.uk@gmail.com and ting010151452@gmail.com, respectively. 

