# Supplementary figures and images for: Regulation of MicroRNA-155 in Atherosclerotic Inflammatory Responses by Targeting MAP3K10
Source: PLoS One. 2012 Nov 26;7(11):e46551. doi: 10.1371/journal.pone.0046551 (PMC3506618; doi:10.1371/journal.pone.0046551)

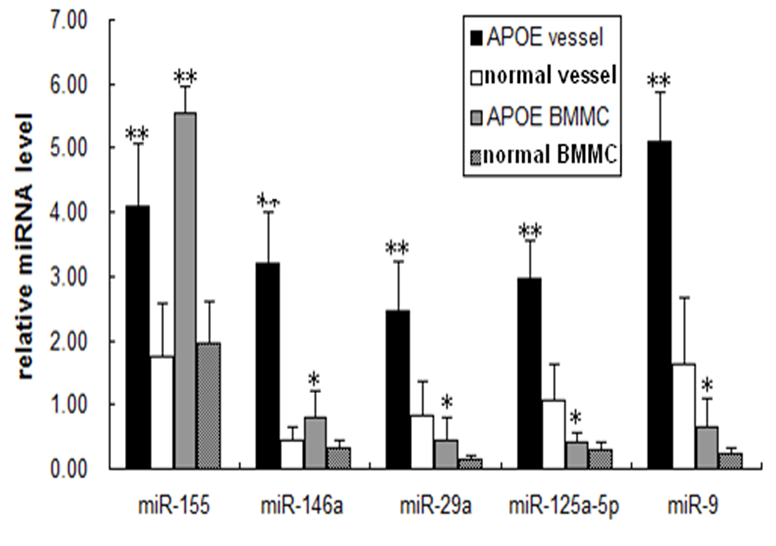

Supplement: Figure S1 — The expression of miRNAs in atherosclerosis mice. Detection of miRNA in vessel and bone-marrow derived mononuclear cell of APOE knockdown mice versus normal mice (n = 5), miRNAs was detected by TaqMan PCR.*P<0.05, **P<0.01. (TIF) [file pone.0046551.s001.tif]

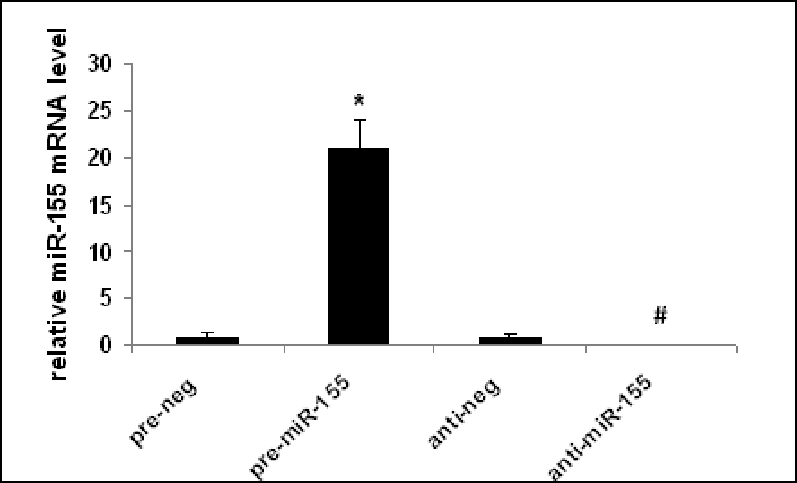

Supplement: Figure S2 — The expression of miR-155 after transfection. Detection the miR-155 expression after transfection with miR-155 mimics and miR-155 inhibitor by TaqMan PCR. (n = 4, *P<0.01, #P<0.01). (TIF) [file pone.0046551.s002.tif]
