# Supplementary material for: Regulation of MicroRNA-155 in Atherosclerotic Inflammatory Responses by Targeting MAP3K10
Source: PLoS One. 2012 Nov 26;7(11):e46551. doi: 10.1371/journal.pone.0046551 (PMC3506618; doi:10.1371/journal.pone.0046551)
Supplement: Table S1 — Characteristics of study cohort. TC, total cholesterol; TG, total glyceride; HDL, high-density lipoprotein; LDL, low-density lipoprotein; WBC, white blood cell; P1: comparison between patients with AMI and without AMI. P2: comparison among patients without and with CHD, Data are presented as means (±SD) or number (%). (DOCX) [file pone.0046551.s004.docx]

**Table S1 Characteristics of study cohort.** TC, total cholesterol; TG, total glyceride; HDL, high-density lipoprotein; LDL, low-density lipoprotein; WBC, white blood cell; P1: comparison between patients with AMI and without AMI. P2: comparison among patients without and with CHD, Data are presented as means (±SD) or number (%).

| Characteristics | | Patients without CAD (n =33) | Patients with CAD | | P1 | P2 |
| --- | --- | --- | --- | --- | --- | --- |
|  |  |  | Non-AMI (n 17) | AMI (n 5 16) | |  |
| Age (years) | | 59.1±8.9 | 62±10 | 66±9.5 | 0.082 | 0.179 |
| Gender Male | | male（100%） | male（100%） | male（100%） | 0 | 0 |
| Current smoking, n (%) | | 13/23 | 15/27 | 25/33 | 0 | 0 |
| Hypertension, n (%) | | 13 | 15 | 19 | 0 | 0 |
| History of | |  |  |  | 0 | 0 |
| AMI/PCI/ACVB/PTA/Stroke | | 0 | 5 | 9 | 0 | 0 |
| Number of vessels(CAD) | | |  |  |  |  |
| 1 |  | 0 | 10 | 9 | 0 | 0 |
| 2 |  | 0 | 9 | 11 | 0 | 0 |
| 3 |  | 0 | 8 | 13 | 0 | 0 |
| TC (mmol/L) | | 1.24±0.3 | 1.77±1.4 | 1.19±0.5 | 0.918 | 0.5 |
| TG (mmol/L) | | 4.14±1.1 | 4.07±1.08 | 4±0.97 | 0.217 | 0.47 |
| HDL (mmol/L) | | 1.12±0.3 | 0.97±0.25 | 0.997±0.25 | 0.085 | 0.209 |
| LDL (mmol/L) | | 2.03±0.76 | 0.96±0.71 | 2.1±0.69 | 0.287 | 0.313 |
| VLDL (mmol/L) | | 0.88±0.3 | 1.17±0.77 | 0.9±0.32 | 0.869 | 0.6 |
| WBC (*109/L) | | 6.16±1.63 | 6.75±1.84 | 9.3±2.24 | 0.193 | 0.283 |
