# Supplementary material for: Regulation of MicroRNA-155 in Atherosclerotic Inflammatory Responses by Targeting MAP3K10
Source: PLoS One. 2012 Nov 26;7(11):e46551. doi: 10.1371/journal.pone.0046551 (PMC3506618; doi:10.1371/journal.pone.0046551)
Supplement: Table S2 — Plasma Cholesterol Levels of APOE-/- mice injected with agomir control and agomir-155 *P<0.05 n = 12. (DOCX) [file pone.0046551.s005.docx]

**Table S2 Plasma Cholesterol Levels of APOE-/- mice injected with agomir control and agomir-155** ***P<0.05 n=12**

|  | agomir control | agomir-155 |
| --- | --- | --- |
| TC, mmol/L | 25.1±2 | 15.6±2.5* |
| Triglycerides, mmol/L | 1.5±0.16 | 1.36±0.24 |
| HDLcholesterol, mmol/L | 2.8±0.31 | 2.4±0.26 |
| LDLcholesterol, mmol/dL | 15.8±2.3 | 6.8±2.1* |
