# Supplementary material for: Regulation of MicroRNA-155 in Atherosclerotic Inflammatory Responses by Targeting MAP3K10
Source: PLoS One. 2012 Nov 26;7(11):e46551. doi: 10.1371/journal.pone.0046551 (PMC3506618; doi:10.1371/journal.pone.0046551)
Supplement: File S1 — Supplementary methods and figures. (DOC) [file pone.0046551.s006.doc]

**Methods**

***Cell culture***

The human monocytic cell line THP-1 was obtained from the American Type Culture Collection (ATCC, Rockville, MD, USA). This was maintained at a density of 106/ml in the RPMI 1640 medium supplemented with 10% fetal bovine plasma, 10 mM HEPES (Sigma), 1% pen/strep solution and placed in a 5% CO2 incubator at 37 °C. To induce monocyte differentiation to macrophage, THP-1 cells were cultured with 100 nM PMA (Calbiochem, San Diego, CA) for 24 h [1].

***In Vivo Assay***

C57BL/6J mice were purchased from Shanghai Institutes for Biological Sciences (shanghai, China), ApoE^-/-^ mice from BeiJing Xie He medicine college (BeiJing, China). ApoE^-/-^ male mice were second- or third-generation hybrid 129olaxC57BL/6 derived from brother-sister mating using only ApoE deficient animals that were initially created by homologous recombination in embryonic stem cells. The ApoE^-/-^ mice (n=24) were fed high-cholesterol diet and examined at ages from 12 weeks. ApoE gene knockout mice showed symptoms of high blood lipid abnormalities, in the 3-month-old when the arteries that is the accumulation of fat, then divided two group(n=12), one group were injected by mismatched miR-155 and the other one were injected by miR-155 .

The miR-155 agomiR was chemically modified and cholesterol conjugated from a hydoxyprolinol-linked cholesterol solid support and 2’-OMe phosphoramidites. For negative control experiments, a mismatched miR-155 agomiR was also synthesized. For delivery of cholesterol conjugated miR-155 agomiR, 10 nmol miR-155 agomiR and mismatched miR-155 agomiR in 0.1 ml saline buffer were tail vein injected once every 3 days for 2 weeks, respectively. Bone marrow-derived dendritic cells were generated from mice as described previously [2], In brief, femurs and tibia of mice were removed and the bone marrow was flushed out with phosphate buffered saline (PBS). The red blood cell (RBC) was removed by incubation with NH4Cl lysing buffer. All animal were approved by the Animal Care and Ethics Committee of the University of Zhe Jiang.

***Small RNA transfection***

MiR-155 mimics and inhibitor were transfected into PMA-induced THP-1 macrophage using the fast transfection protocol recommended for Hiperfect transfection reagent (Qiagen) at a final concentration of 50 nM. Cells were incubated with oxLDL (50ug/ml) for 24 h and then transfected with miRNA mimic/inhibitor for 24 h. To confirm the efficiency of transfection, the same amount of Cy3-labeled negative control was also transfected. SiMAP3K10 and siNS (not significant) duplex were synthesized by Dharmacon RNAi Technologies. miRNA and mRNA Real Time Quantitative PCR .To detect miRNAs from cells, tissues and plasma, total RNA was isolated using the miRVana miRNA isolation kit (Ambion) according to the protocol of the manufacturers. Total and miRNA-specific cDNA was generated with TaqMan® MicroRNA Reverse Transcription Kit (Ambion), and miRVana quantitative RT-PCR primer sets for miRNAs (Ambion). The PCR reaction was directly monitored by the ABI PRISM 7000 Sequence Detection System (Applied Biosystems, CA). Briefly, cDNA was made from enriched miRNA using the TaqMan MicroRNA RT Kit. U6 RNA was used as an endogenous control.

The mRNA levels were analyzed using the SYBR-GREEN reagent kits on the Applied Biosystems 7000 real-time PCR system. Human glyceraldehyde 3-phosphate dehydrogenase (GAPDH) was also amplified to serve as an internal standard. The miRNA mimics and inhibitor sequences are listed as follow, and the efficiency of the transfection is shown as the online figure1:

hsa-miR-155 mimics：(5'to 3') F: UUAAUGCUAAUCGUGAUAGGGGU

(5'to 3') R: ACCCCUAUCACGAUUAGCAUUAA

hsa-miR-155 inhibitor： ACCCCUAUCACGAUUAGCAUUAA

2’Ome modification for each one

mimics control: (5'to 3') F: UCACAACCUCCUAGAAAGAGUAGA

(5'to 3') R: UCUACUCUUUCUAGGAGGUUGUGA

Inhibitor control : (5'to 3') F: UCUACUCUUUCUAGGAGGUUGUGA

2’Ome modification for each one

***ELISA assays of inflammatory markers***

Cell or plasma supernatants were analyzed to determine the presence of TNF-α, IL-6 using Sandwich Enzyme Immunoassay kits (R&D) according to the manufacturer's instructions.

***Western blot analysis***

Protein extracts were denatured and the solubilized proteins (20 μg) were subjected to electrophoresis on 10% polyacryl amide SDS gels. This was followed by probing with antibodies (Abcom) for rabbit anti-human-MAP3K10, JNK, P38, ERK1/2 (diluted 1:1000 in TBST), or rabbit anti-actin (diluted 1:5000 in TBST), and then by goat anti-rabbit secondary antibody labeled with far-red-fluorescent Alexa Fluor 680 dye. All signals were detected using the Odyssey system (Li-cor, USA). Densitometric analysis was performed using Quantity One (Bio-Rad) to scan the signals.

***Cloning of 3′ UTR of MAP3K10 mRNA and Reporter Gene Assay***

The THP1 cell were cotransfected with a p-MAP3K10 UTR and mut-MAP3K10 UTR miRNA luciferase reporter vector and miR-155 inhibitor using lipofectamine 2000 (Invitrogen). Cells were also transformed with the PGL3-control vector, which is useful for monitoring transfection efficiency. Anti-miR negative, an miRNA non-homologous to the human genome, was used as a control. All cells were also transfected with pRL-TK (Promega) as a normalization control. After 24 h, firefly luciferase activity was determined using the dual-luciferase reporter assay system. Relative reporter activity was obtained by normalization to the Renilla control.

***Analysis of Atherosclerotic Plaque Size and Composition***

After two weeks, mice were anesthetized with intraperitoneal injection of ketamine (60mg/kg) and xylazine (6mg/kg) and then mice were killed by cervical dislocation as soon as possible. Plasma cholesterol was measured with a commercially available cholesterol kit (Sigma). Morphometric and immunohistochemical studies were performed in the aortic sinus and the thoracic aorta (spanning from the brachiocephalic artery to the renal arteries and including the first 3 mm2 of the brachiocephalic artery). Lesion size and composition were assessed as described previously [3]. Serial sections were taken throughout the entire aortic valve area and routinely stained with hematoxylin and eosin. To quantitate plaque size, images of 3 sections were acquired with a DP70 digital camera connected to a microscope (Olympus), and lesion areas determined using Image-Pro plus v 6.0 software (Media Cybernetics Inc). The mean value of plaque cross-sectional areas from 3 sections was used to estimate the extent of atherosclerosis for each animal.

Immunohistochemical analysis was performed as described previously[4]. The following primary antibodies were used: anti-mouse CD68 (Santa Cruz), anti–MAP3K10 (Sigma), anti–TNF-a (Santa Cruz) and anti–IL-6 antibody (Santa Cruz). At least 4 sections per animal were analyzed for each immuno- staining.

***Collection and storage of plasma and peripheral blood mononuclear cells (PBMCs)***

Blood samples for miRNA detection were collected from the patients and in the emergency department or cardiac catheterization laboratory. The samples were processed within 1 h of collection by two-step centrifugation. Peripheral blood mononuclear cells were isolated from the fresh blood of patients using Ficoll–Hypaque.

**References**

[1]. Tsuchiya S, Kobayashi Y, Goto Y et al. (1982) Induction of maturation in cultured human monocytic leukemia cells by a phorbol diester. Cancer Res 42:1530–6.

[2].Son, Y.I., Egawa, S., Tatsumi, T., Redlinger Jr., R.E., Kalinski, P et al. A novel bulk-culture method for generating mature dendritic cells from mouse bone marrow cells. (2002) J. Immunol. Method 262: 145–157.

[3]. Paigen B, Morrow A, Holmes PA, Mitchell D, Williams RA (1987) Quantitative assessment of atherosclerotic lesions in mice. Atherosclerosis. 68: 231–240.

[4]. Sukhanov S, Higashi Y, Shai SY, Vaughn C, Mohler J et al. (2007) IGF-1 reduces inflammatory responses, suppresses oxidative stress, and decreases atherosclerosis progression in ApoE^-/-^ mice. Arterioscler Thromb Vasc Biol 27(12):2684-90.
